# Potentiation of Morphine-Induced Antinociception by Propranolol: The Involvement of Dopamine and GABA Systems

**DOI:** 10.3389/fphar.2017.00794

**Published:** 2017-11-10

**Authors:** Elham A. Afify, Najlaa M. Andijani

**Affiliations:** ^1^Department of Pharmacology, Faculty of Pharmacy, King Abdulaziz University, Jeddah, Saudi Arabia; ^2^Department of Pharmacology and Toxicology, Faculty of Pharmacy, Alexandria University, Alexandria, Egypt

**Keywords:** opioids, propranolol, antinociception, hot plate, formalin, acetic acid, D_2_ receptors, GABA_A_ receptors

## Abstract

Tolerance to the analgesic effect of morphine is a major clinical problem which can be managed by co-administration of another drug. This study investigated the ability of propranolol to potentiate the antinociceptive action of morphine and the possible mechanisms underlying this effect. Antinociception was assessed in three nociceptive tests (thermal, hot plate), (visceral, acetic acid), and (inflammatory, formalin test) in mice and quantified by measuring the percent maximum possible effect, the percent inhibition of acetic acid-evoked writhing response, and the area under the curve values of number of flinches for treated mice, respectively. The study revealed that propranolol (0.25–20 mg/Kg, IP) administration did not produce analgesia in mice. However, 10 mg/Kg propranolol, enhanced the antinociceptive effect of sub-analgesic doses of morphine (0.2, 1, and 2 mg/Kg, IP) in the three nociceptive tests. It also shifted the dose response curve of morphine to the left. The combined effect of propranolol and morphine was attenuated by haloperidol (D_2_ receptor antagonist, 1.5 mg/Kg, IP), and bicuculline (GABA_A_ receptor antagonist, 2 mg/Kg, IP). Repeated daily administration of propranolol (10 mg/Kg, IP) did not alter the nociceptive responses in the three pain tests, but it significantly potentiated morphine-induced antinociception in the hot plate, acetic acid-evoked writhing, and in the second phase of formalin tests. Together, the data suggest that a cross-talk exists between the opioidergic and adrenergic systems and implicate dopamine and GABA systems in this synergistic effect of morphine-propranolol combination. Propranolol may serve as an adjuvant therapy to potentiate the effect of opioid analgesics.

## Introduction

Pain is an important reflex that warns against a potential damage or injury, and has been the subject of intense study and research. Until today, morphine is believed to be the most effective pain killer. However, its use is hampered by the development of tolerance, dependence and respiratory depression (Garcely, 1995; Sehgal et al., 2013). Catecholaminergic system has a pivotal role in regulating the opioid activity. Local and subcutaneous application of β-blockers has antinociceptive effect. For example, blockade of β-adrenoceptors in the joints connecting the jawbone to the skull induced antinociception in experimental animals (Fávaro-Moreira et al., 2012). In clinical studies, esmolol (Chia et al., 2004; Collard et al., 2007; Haghighi et al., 2015), atenolol (Zaugg et al., 1999), and labetalol (Xiao et al., 2008) demonstrated analgesic properties and reduced the recurrent postoperative pain. Coloma et al. (2001) suggested that perioperative β-antagonist administration was an alternative to opioids to maintain postoperative analgesia. Additionally, non-selective β-adrenergic receptor blockers inhibited the development of morphine tolerance in mice (Kihara and Kaneto, 1986; Kaneto and Inoue, 1990); reduced naloxone precipitated opioid withdrawal and might be effective in the treatment of opiate addiction (Harris and Aston-Jones, 1993). However, the specific mechanisms by which β-blockers potentiate the analgesic effect of opioids remains controversial.

Propranolol is one of the prototype clinically prescribed cardioprotective β-adrenergic receptor blockers primarily used in treatment of arrhythmia and hypertension (Degoute, 2007; Koelemay and Legemate, 2008). It showed cutaneous analgesia due to membrane stabilizing activity, either alone (Chen et al., 2012) or combined with dopamine (Chen et al., 2015) against nociceptive stimuli in rats. It is reasonable that in certain painful situations propranolol can modulate nociceptive signals and may be effective on occasion as a combined analgesic medication (Smith, 2006). These reported studies evaluated the effect of propranolol on cutaneous skin insults. Whether propranolol administered systemically will show some promise as an agent to enhance morphine analgesia has not been studied.

In this study we explored the ability of propranolol to modify the analgesic response to morphine and the possible mechanisms underlying this effect in three models of pain. To this purpose, morphine and propranolol were administered alone or combined to female mice. To assess antinociception, the hot plate, acetic acid-evoked writhing, and formalin tests were employed and quantified by measuring the percent maximum possible effect, the percent inhibition of acetic acid-evoked writhing and the area under the curve values of formalin-induced flinches for treated mice. Additionally, pharmacological antagonists were used to define the possible role of dopamine and GABA receptors in the antinociceptive effect of propranolol-morphine combination in mice.

## Materials and Methods

### Animals

Female Swiss mice (30–40 g: Animal care facility of King Fahd Medical Research Center, King Abdulaziz University in Jeddah) were employed in the present study. Mice were housed in cages under standard conditions (temperature: 22 ± 2°C, 12/12 h light/dark cycle, free access to water and standard chow). All experiments were approved by the institutional research unit of the biomedical ethics for the care and handling of experimental animals (Reference No. 278–17). The experimental animals were utilized for one behavior test and were sacrificed under anesthesia at the end of the experiment. The observer of the performed experiments was blind to the treatment type.

### Drugs

The following drugs were used: Morphine sulfate (10 mg/ml) (Laboratoire Renaudin, Saint-Cloud, France), propranolol (Fluke, Chemie GmbH, Basel, Switzerland), formaldehyde solution (Riedel-de Haën, Seelze, Germany), bicuculline (Sigma Chemical Co., St Louis, MO, United States), and haloperidol (Jamjoom pharma factory, Jeddah, Kingdom of Saudi Arabia). Drugs were dissolved in 0.9% NaCl. Two drops of concentrated acetic acid 97% was added to bicuculline solution for stability purpose (Hemnani et al., 1983).

### Experimental Design

**FIGURE 1:**
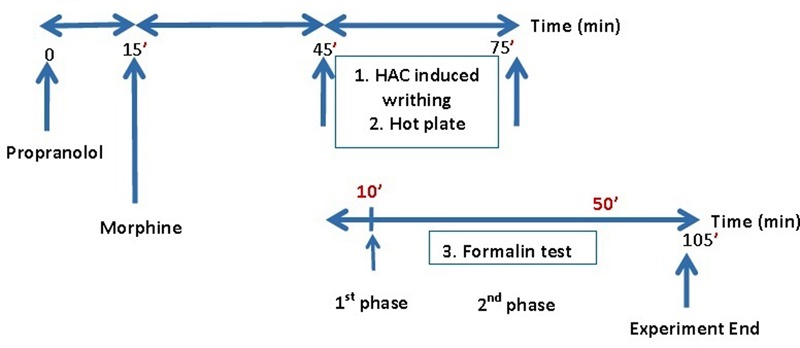
The treatment regimen in different pain models.

Propranolol was administered intraperitoneally (IP) at doses of 0.5–20 mg/Kg in mice. The 10 mg/Kg dose of the drug was then used for all of the subsequent experiments. Morphine was administered at 0.2–8 mg/Kg, IP and propranolol was administered 15 min before morphine, which was administered 30 min prior to the pain test. Control mice were injected with saline and the drugs were administered to the mice with the total volume of 0.1 mL/10 g of body weight. **Figure 1** illustrates the treatment regimen in the pain models.

### Acetic Acid-Evoked Abdominal Writhing

After treatment with propranolol or morphine, mice (*n* = 6–8/group) were administered acetic acid solution (0.6%, 10 mL/kg, IP). Injection of acetic acid produced typical abdominal contractions in mice in the form of waves of muscles contractions accompanied by extension of the hind limb. The potency of the nociceptive stimulus was evaluated by recording the number of writhes for 30 min following acetic acid injection and the percentage of inhibition of acetic acid-evoked writhing was quantified according to Koster et al. (1959) using the following formula:

$$\%\text{ inhibition}=\frac{\left[\text{Writhes in the control mice}-\text{Writhes in post drug mice}\right]}{\text{Writhes in control mice}}\times 100$$

### Hot Plate Test

The hot plate apparatus (Ugo Basile Comerio, Italy) was heated at 50 ± 1°C. Thirty minutes after morphine administration, each mouse was positioned on the preheated metallic base of the apparatus till the appearance of the painful symptoms as lifting or licking of the hind paws or escaping out the chamber. The time in seconds between placing the mouse on the hot surface of the plate and the appearance of nociceptive signs was recorded using stop watch with a cut off time of 30 s as described by Woolfe and MacDonald (1944). Antinociception was quantified as percent maximum possible effect (%MPE) induced by the drugs according to the equation:

$$\%\text{MPE}=\frac{\left(\text{Latency of test group}-\text{Latency of control group}\right)}{\left(\text{Cut off}-\text{Latency of control group}\right)}\times 100$$

### Formalin Test

The test was done by introducing the mice in 25 cm × 15 cm × 20 cm translucent plastic cage for a period of 30 min to get used to the surroundings provided in the experiment. A 27-gauge needle was used to inject 50 μl of 1% formalin solution into the plantar exterior of the right hind paw of the mice. The Mice were then positioned in the cage, and pain associated behaviors were evaluated and analyzed by calculating the frequency of the flinching of the injected paw in which the solution was injected (Hunskaar and Hole, 1987). Flinches were calculated and analyzed for a whole 60 min after IP injection at intervals of 5 min. Intraplantar injections of formalin solution elicited a biphasic reaction. The first phase started immediately following formalin injection, lasted for 5–10 min and indicated acute nociceptive response. The second phase referred as the prolonged tonic phase started 10–15 min subsequent to the formalin injection and its effect remained evident till 60 min. For comparative reason, graphical presentations were created for the AUC of the number of flinches in opposition to the time period by the Graph Pad Prism Version 5.2 for the Windows based on the trapezoidal rule.

Further, mechanisms by which propranolol potentiated morphine antinociception in mice in the three pain tests were performed using antagonists. For each pain test, eight groups of mice were used. I: (Control group) injected with equal volumes of 0.9% NaCl, II: propranolol (10 mg/Kg, IP), III: morphine (dose range: 0.2–4 mg/Kg according to pain test), IV: propranolol + morphine, V: haloperidol (1.5 mg/Kg, IP) as dopamine D_2_ receptor antagonist, VI: bicuculline (2 mg/Kg, IP) as GABA_A_ receptor antagonist, VII (propranolol/morphine/haloperidol), and Group VIII (propranolol/morphine/bicuculline). Doses of blockers were chosen based on previous work done in our lab and by others (Omar, 2007; Afify et al., 2017). Treatment with blockers was started 15 min prior to IP administration of propranolol. Propranolol was administered 15 min before morphine, which was administered 30 min prior to the nociceptive test. The antinociceptive response was measured for 30 min (hot plate, acetic acid-evoked writhing) or 60 min (formalin test) after morphine treatment as previously described.

### Repeated Daily Administration Experiments

Since the 10 mg/Kg dose of propranolol potentiated the antinociceptive effect of morphine in the three pain models, it was appreciated to test this dose further to investigate the effect of repeated administration of propranolol. Mice were divided into four groups in each pain test and injections started at 8AM for four consecutive days as follows: I: (Control group) received equal volumes of the vehicle (saline), II: propranolol (10 mg/Kg, IP), III: morphine (0.2 mg/Kg, IP), IV: propranolol-morphine combination. Propranolol was administered at zero time and followed by, 15 min later, morphine and animals were tested 30 min after morphine. On the 4th day, the antinociceptive response was measured for 30 or 60 min after morphine treatment according to the pain test, as stated previously.

### Statistical Analysis

Values are presented as mean ± SEM Data were analyzed by one-way analysis of variance (ANOVA) followed by Bonferroni’s *post hoc* test with the level of significance set at *P* < 0.05 using the Graph Pad Prism (Version 5.2) for Windows.

## Results

### Effect of Propranolol on Morphine-Induced Antinociception in Acetic Acid-Evoked Writhing

**FIGURE 2:**
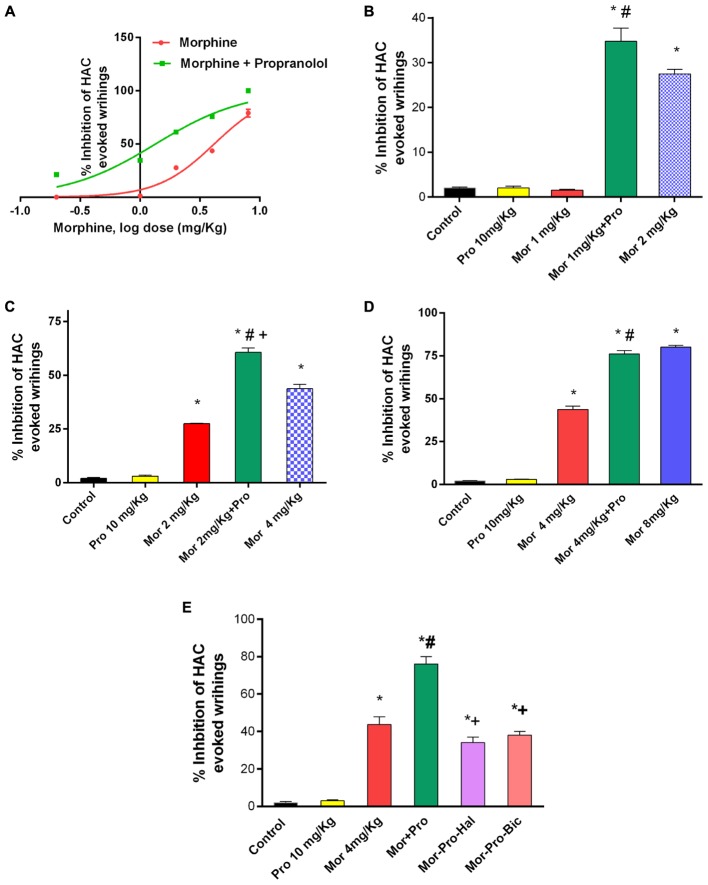
Effects of administration of morphine (Mor) and propranolol [Pro, 10 mg/Kg, intraperitoneally (IP)] either alone or in combination on acetic acid-evoked writhing in mice. **(A)** The dose-response curve for the antinociceptive effect of morphine alone (0.2–8 mg/Kg, IP) and morphine-propranolol combination. **(B–D)** The antinociceptive effect of Mor, Pro and their combination. **(E)** The effect of pretreatment with the dopamine receptor antagonist haloperidol (Hal, 1.5 mg/Kg, IP) and GABA_A_ receptor antagonist bicuculline (Bic, 2 mg/Kg, IP) on the antinociceptive effect of morphine-propranolol combination. Each point represents the mean of % inhibition of acetic acid-evoked writhing ± SE for 6–8 mice. ^∗^*P* < 0.05 compared with control, ^#^*P* < 0.05 compared with Mor 1 mg/Kg **(B)**, Mor 2 mg/Kg **(C)**, Mor 4 mg/Kg **(D,E)**, ^+^*P* < 0.05 compared with Mor-Pro group, by one-way ANOVA and Bonferroni’s *post hoc* test.

Acetic acid injection to mice (0.6%, 0.1 mL/10 g) produced a typical abdominal constriction-stretching response between 0 and 30 min. Morphine dose dependently caused inhibition of the abdominal constrictions but propranolol (0.25–20 mg/Kg, IP) failed to elicit antinociceptive response compared to saline control group. IP administration of sub-antinociceptive doses of morphine (0.2, 1, and 2 mg/Kg) resulted in antinociception levels that does not exceed 27.5% inhibition of HAC evoked writhing. By contrast, mice receiving morphine and propranolol showed a significantly enhanced inhibition of HAC evoked writhing compared to mice treated with morphine alone and saline control (*P* < 0.05). Propranolol caused a parallel leftward shift in morphine dose response curve (ED50 = 1.342 mg, vs. 4.119 mg, for morphine group) (**Figure 2A**). The antinociception obtained with 10 mg/Kg propranolol plus 1 mg/Kg morphine was statistically not different from that obtained with 2 mg/Kg morphine administered alone to mice (%HAC inhibtion were 34.75 and 27.5%), respectively, (**Figure 2B**). The antinociception obtained with 10 mg/Kg propranolol combined with 2 mg/Kg morphine was higher than that obtained with 4 mg/Kg morphine administered alone to mice (%HAC inhibtion were 60.67 and 43.70%), respectively, (**Figure 2C**). Similarily, the antinociception obtained with 10 mg/Kg propranolol plus 4 mg/Kg morphine was equal to that induced by 8 mg/Kg of morphine administered alone (%HAC inhibtion were 76% and 80%), respectively, (**Figure 2D**). Moreover, IP administration of morphine (8 mg/Kg) plus propranolol (10 mg/Kg) to mice resulted in maximum possible antinociception (100% inhibition of HAC evoked writhing) compared to 80% in mice treated with morphine alone. The effect of administration of haloperidol and bicuculline was studied using HAC evoked writhing response (**Figure 2E**). One-way ANOVA showed a significant effect of treatment [*F*(5,24) = 101.6, *P* < 0.0001]. The antinociceptive response of morphine-propranolol combination was significantly reduced by haloperidol or bicuculline treatment (% inhibition of HAC induced writhing were 76 ± 4, 34 ± 3, and 38 ± 2, respectively. Levels of antinociception observed in haloperidol or bicuculline treated groups were not significantly different from the corresponding values obtained in saline control group (data not shown).

### Effect of Propranolol on Morphine-Induced Antinociception in the Hot Plate Test

**FIGURE 3:**
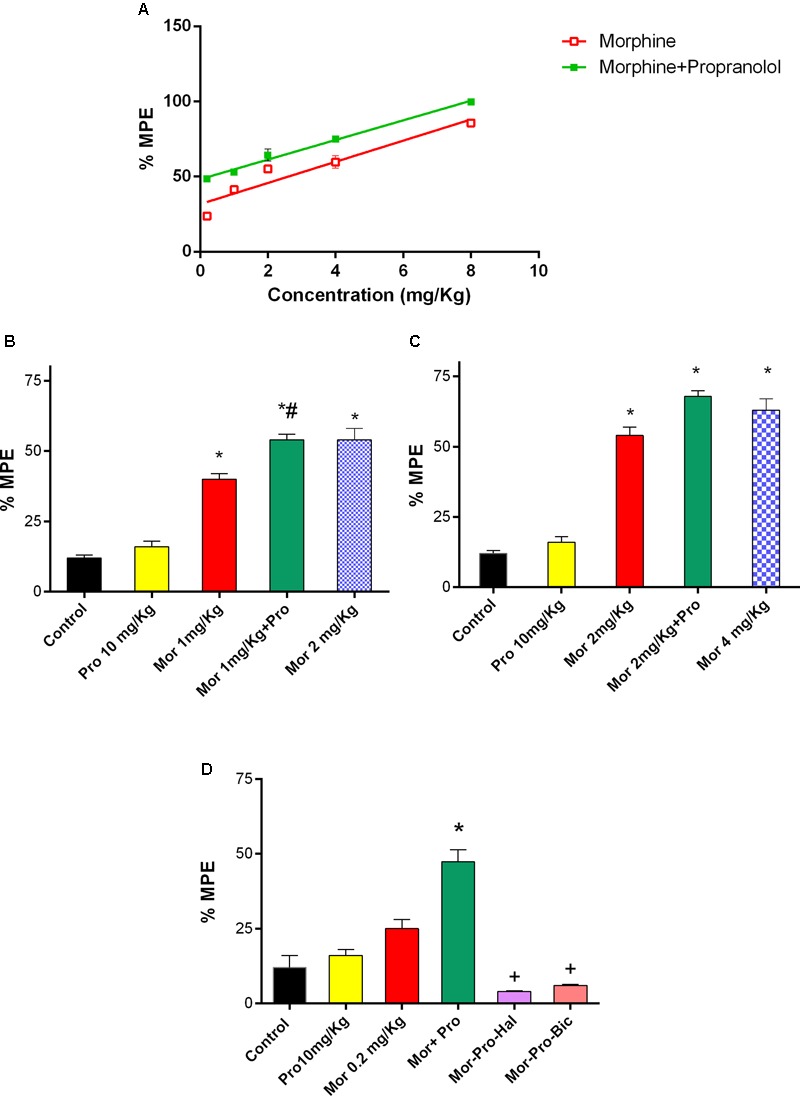
Effect of administration of propranolol (Pro, 10 mg/Kg, IP) on the antinociceptive effect of morphine (Mor) in the hot plate test represented by percent maximum possible effect (%MPE). **(A)** The dose–response curve for the antinociceptive effect of morphine alone (0.2–8 mg/Kg, IP) and morphine-propranolol combination. **(B,C)** The antinociceptive effect of different doses of morphine, propranolol and their combination. **(D)** The effect of pretreatment with dopamine receptor antagonist haloperidol (Hal, 1.5 mg/Kg, IP) and bicuculline, GABA_A_ receptor antagonist (Bic, 2 mg/Kg, IP) on the antinociceptive effect of morphine-propranolol combination. Each point indicated the mean %MPE ± SE for 6–8 mice. ^∗^*P* < 0.05 compared with control, ^#^*P* < 0.05 compared with Mor 1 mg/Kg, ^+^*P* < 0.05 compared with Mor-Pro group, by one-way ANOVA and Bonferroni’s *post hoc* test.

Mice treated with saline did not exhibit antinociceptive response using the hot plate test. Propranolol (0.25–20 mg/Kg, IP) did not produce significant antinociceptive response (*P* > 0.05). On the other hand, morphine (0.2–8mg/Kg, IP) produced a dose-dependent antinociception (*F* = 34.73, *P* < 0.05, **Figure 3A**). Although the lowest tested dose of morphine (0.2 mg/Kg, IP) did not induce a signifcant antinociception, the combination of propranolol (10 mg/Kg, IP) and morphine (0.2–8 mg/Kg, IP) induced a dose-dependent antinociceptive response [*F*(5,24) = 105.4, *P* < 0.05]. Animals receiving 10 mg/Kg propranolol and 1 mg/Kg morphine (%MPE 54.12 ± 2) showed a significant increase in antinociceptive response compared to 1 mg/Kg morphine given alone and saline control groups (*P* < 0.05). The response was comparable to that induced by 2 mg/Kg of morphine given alone (**Figure 3B**). Similarily, combined administration of 10 mg/Kg propranolol and 2 mg/Kg morphine produced effect similar to that produced by 4 mg/Kg morphine administered alone (**Figure 3C**). In the receptor antagonism tests, one way ANOVA showed a significant effect of treatment [*F*(5,24) = 34.27, *P* < 0.001, **Figure 3D**]. Antinociception induced by combined propranolol (10 mg/Kg) – morphine (0.2 mg/Kg) therapy was abolished by haloperidol (%MPE = 4 ± 0.3) or bicuculline treatment (%MPE = 6 ± 0.3). The %MPE was not affected by treatment with either haloperidol or bicuculline alone compared with saline control group (data not shown).

### Propranolol Potentiated Morphine-Induced Antinociception in Both Phases of Formalin Test

**FIGURE 4:**
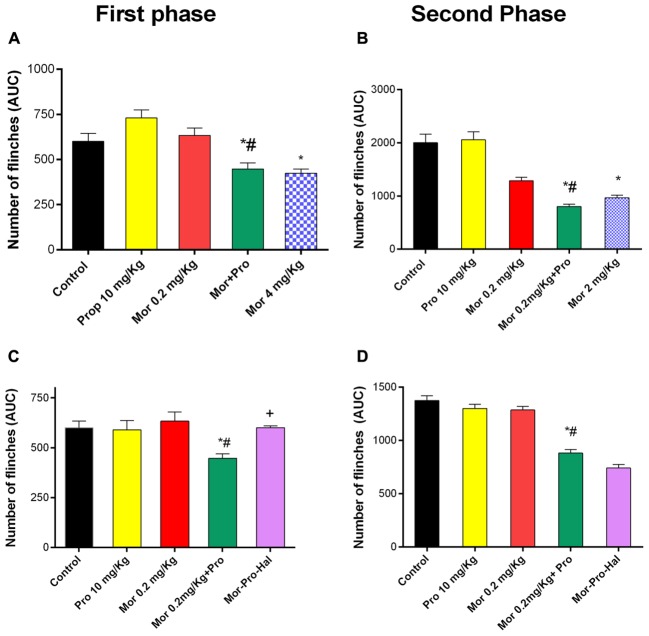
Effect of administration of morphine (Mor, 0.2 mg/Kg, IP) and propranolol (Pro, 10 mg/Kg, IP) on the hind paw flinches quantified during the two phases of the formalin test in mice during the first phase **(A)** and second phase **(B)** of the test. The effect of pretreatment with haloperidol, dopamine receptor antagonist (Hal, 1.5 mg/Kg, IP) on the antinociceptive effect of morphine-propranolol combination during both first and second phases **(C,D)**, respectively. Each point represents the mean area under the curve (AUC) for the number of flinches ± SE for 6–8 mice. ^∗^*P* < 0.05 compared with control, ^#^*P* < 0.05 compared with Mor 0.2 mg/Kg, ^+^*P* < 0.05 compared with Mor-Pro group, by one-way ANOVA and Bonferroni’s *post hoc* test.

Intraplantar injection of 1% formalin to the mice evoked a typical biphasic flinching response. The AUC of the number of flinches for the first phase (0–15 min) was 600 ± 45 and for the second phase (15–60 min) was 2000 ± 160 in control group. Administration of morphine (0.2 mg/Kg) or propranolol (10 mg/Kg) had no effect on pain response as evident by insignificant changes in the AUC of number of flinches of either first (633 ± 40, 730 ± 40, and 600 ± 45) or second phase (1285 ± 67, 2056 ± 150, and 2000 ± 160) of the formalin test compared with saline control (*P* > 0.05, **Figures 4A,B**). Propranolol-morphine combination significantly reduced the number of flinches during the first phase of formalin test compared to saline control. Animals receiving 10 mg/Kg propranolol and 0.2 mg/Kg morphine showed a significantly enhanced antinociceptive response compared to 0.2 mg/Kg morphine alone and saline control treated mice (447 ± 34, 633 ± 40, and 600 ± 45), respectively, (*P* < 0.05). The response was comparable to that induced by 4 mg/Kg of morphine (423 ± 23) given alone (**Figure 4A**). Similar potentiation of morphine reponse was observed during the second phase of formalin test (**Figure 4B**). Administration of haloperidol significantly antagonized the antinociceptive effect of propranolol-morphine combination in the first but not the second phase of the test. The number of flinches during the first phase was 600 ± 10 vs. 447 ± 23 for morphine-propranolol group (**Figure 4C**, *P* < 0.05) and second phase was 740 ± 34 vs. 880 ± 34 for morphine-propranolol group (**Figure 4D**, *P* < 0.05).

### Repeated Daily Administration of Propranolol Potentiated Morphine-Induced Antinociception in the Three Nociceptive Tests

In the acetic acid-evoked writhing, hot plate, and the formalin tests, mice treated with propranolol 10 mg/Kg IP for 4 days did not exhibit antinociceptive response compared to saline treated group (**Table 1**). However, combined treatment with propranolol and morphine for 4 days significantly potentiated morphine induced antinociception in the three pain tests. The % inhibition of writhes of propranolol-morphine combination was 71%, *P* < 0.05. **Table 1** indicated that animals treated with morphine-propranolol combination exhibited significant increase in % MPE of 26. 30 ± 3.40 compared to 2 ± 0.05 for the saline control group (*P* < 0.05) in the hot plate test. In the second phase of formalin test, combined treatment with propranolol-morphine had significant antinociceptive effect (39.03% inhibition, *P* < 0.05). The AUC for the number of flinches of morphine-propranolol combination were 550.5 ± 31.30 in the first phase and 838.1 ± 82.22 in the second phase compared to 477.5 ± 30 and 1374.62 ± 57 for the saline control group, respectively.

**Table 1:** Effect of repeated daily treatment with propranolol and morphine on the antinociceptive responses of mice.

Treatment	Acetic acid-evoked writhing	Hot plate test	Formalin test	
	No. of abdominal writhing	%MPE	AUC of the flinches number	
			First phase	Second phase
Saline (Control)	106.66 ± 7.98	2 ± 0.05	477.5 ± 30	1374.62 ± 57
Propranolol	102.2 ± 8	4 ± 0.60	535 ± 28	1400 ± 67
Morphine	96 ± 6	1.8 ± 0.30	450 ± 45	1220 ± 78
Propranolol + Morphine	31.40 ± 4.33^∗^ (**71%**)	26.30 ± 3.40^∗^	550.5 ± 31.30 (**13.26%**)	838.1 ± 82.22^∗^ (**39.03%**)

*Propranolol (10 mg/Kg), morphine (0.2 mg/Kg) were administered intraperitoneally (IP) for 4 days to mice. On the 4th day of experiment mice were subjected to the assigned nociceptive test. **Acetic acid test:** Abdominal writhing responses to IP injections of acetic acid (0.6% solution, 10 mL/Kg) were counted for 30 min and the % inhibition of acetic acid-evoked writhing was quantified. **Hot plate:** Each mouse was placed on a metallic surface of the hot plate after 30 min of injections with morphine (0.2 mg/Kg, IP), the latency in seconds was recorded as the time between placement of the animal on the hot plate and the occurrence of symptoms of discomfort as licking of the hind paws, shaking or jumping off from the surface with a cut-off time of 30 s, hot plate latency data were expressed as percent maximum possible effect (%MPE). **Formalin test:** 50 μl of 1% formalin was injected into the plantar surface of the right hind paw with a 27-gauge needle and the number of flinches of the injected paw was quantified for a total of 60 min after intraplantar injection at 5-min interval. The first phase began immediately after formalin injection and lasted for 5–10 min. The second phase began 10–15 min after formalin injection and lasted for 50 min. The area under the curve (AUC) of the flinches number against time was calculated. Results are Mean ± SEM (*n* = 6). ^∗^*P* < 0.05 compared with control by one-way ANOVA and Bonferroni’s *post hoc* test. % inhibition between brackets.*

## Discussion

The results of the present study are the first to demonstrate that propranolol; a non-selective β-adrenergic blocker potentiated the effect of sub-analgesic doses of morphine in animal models of thermal, visceral and inflammatory pain. Additionally, it reduced the doses of morphine required to achieve a maximum antinociceptive response as indicated by the reduced ED50 of morphine suggesting a greater effect of the combination therapy. The present study is also the first to implicate GABAergic and dopaminergic systems in the analgesic effect of morphine-propranolol combination. Data obtained from nociceptive experiments concerning the ability of β-blockers to potentiate morphine analgesia are conflicting. Propranolol alone (Chen et al., 2012) or combined with lidocaine or dopamine (Chen et al., 2015) intensified the analgesia against skin nociceptive stimuli in rats. Similar observations were reported for other β-adrenergic antagonists as atenolol (Zaugg et al., 1999), labetalol (Xiao et al., 2008), and esmolol (Chia et al., 2004; Collard et al., 2007; Haghighi et al., 2015). In another study, β_2_-adrenergic receptors modulated both opioid tolerance and physical dependence. Administration of butoxamine reversed morphine tolerance in mice (Liang et al., 2007). Left unclear the ability of propranolol to modify the analgesic effect of sub-analgesic doses of morphine and the mechanisms underlying this effect. In contrast to the observed potentiation of the antinociceptive effect of propranolol-morphine combination in our study, previous studies have shown that propranolol didn’t modify the dose response curve to the antinociceptive action of morphine or alter the ED50 of morphine in the tail flick test (Fennessy and Lee, 1970; Gorlitz and Frey, 1972; Richard et al., 1975). The reason for this discrepancy may be attributed to the difference in the pain model itself or the short time interval between the administration of propranolol and morphine.

Opioid receptors, β-adrenergic receptors and dopamine receptors belong to the G protein coupled receptor family (GPCR) (Zheng et al., 2010) that mediates antinociceptive effects via similar signal transduction pathways. β-adrenergic stimulation positively impacts adenylyl cyclase (AC) and stimulates protein kinase A (PKA) (Pepe et al., 2004; Grimm and Brown, 2010). Opioid receptors acting in an inverse way through G i/o proteins, which blunt the recruitment of PKA via inhibiting (AC) and reducing the level of cyclic adenosine monophosphate (cAMP) (Law and Loh, 1999). Remarkably, reported studies highlighted the role of opioids in facilitating β-adrenergic blockade. That said, a possible additive effect could exsist between blocking of β-adrenergic receptors and stimulation of opioid receptors in reducing cellular cAMP (Vamecq et al., 2015). The inhibition of cAMP signaling pathway alleviates nociceptive sensations in the pain memory (Shao et al., 2016), since the activation of the cAMP/PKA signaling pathway can improve the recognition function and generated hyperalgesia (Li et al., 2015). Moreover, the elevation of cAMP produces nociception in rodent pain models (Dolan and Nolan, 2001; Song et al., 2006). These findings provide evidence that links decreased cAMP to antinociception and increases to blockade of analgesia. Therefore, propranolol by blocking the adrenergic receptors inhibits the stimulatory action of Gs on the AC enzyme, blocks the generation of cAMP, augments the inhibitory effect of opioids on pain transmission and potentiates the antinociceptive response. This in turn supports the crosstalk between propranolol and morphine in potentiating morphine antinociception. In fact the analgesic effect of morphine has been linked to mechanisms more than changes in the level of cAMP such as reducing neurotransmitter release at the presynaptic levels via inhibition of calcium channels (Kumar et al., 2010). Morphine also can hyperpolarize nociceptors at the postsynaptic levels through activation of potassium channels (Khanna et al., 2011). The link between these pathways and β-adrenergic receptors warrants further investigation.

**FIGURE 5:**
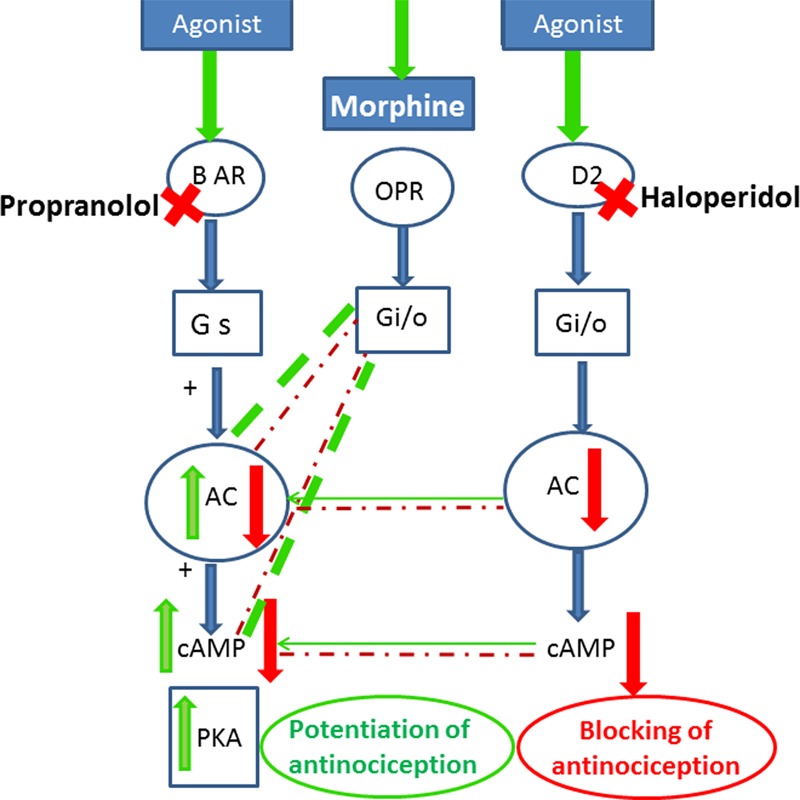
Scheme for cross-talk between opioid, adrenergic and D_2_ dopamine receptors that may lead to the potentiation of opioid antinociception after treatment with propranolol. Stimulatory effects are indicated by green arrows/lines, inhibitory effects in red arrows/lines, the potentiating effects are shown in thick dashed green lines. Agonist stimulation of β-adrenergic receptors activates (AC) and stimulates (PKA). Morphine acting on opioid receptors activates G i/o proteins, which blunts the recruitment of PKA via inhibiting (AC) and reducing the level of cAMP. Blocking of β-adrenergic receptors by propranolol and stimulation of opioid receptors by morphine reduce cellular cAMP and potentiate the antinociceptive response. Blocking of the D_2_ receptors by haloperidol prevents the inhibitory effect of dopamine on AC enzyme and the reduction in cAMP level and antagonizes the inhibitory effect of morphine and propranolol on reducing cAMP level and reversed their antinociceptive effect. BAR, β-adrenergic receptors; ORP, opioid receptors; Gs, stimulatory G-protein; Gi, inhibitory G protein; AC, adenylyl cyclase; cAMP, cyclic adenosine monophosphate; PKA, protein kinase A.

Interestingly, our results indicated that the D_2_ receptor blocker haloperidol antagonized the antinociceptive effect of propranolol-morphine combination. The role of dopamine receptors was widely accepted generally in analgesia and specifically in morphine antinociception. The painful symptoms observed in Parkinson’s disease and fibromyalgia are associated with decreased dopamine levels (Wood, 2008). Recently, it has been shown that D_2_ receptor agonists inhibited allodynic responses in rats (Cobacho et al., 2014) and mice (Almeida-Santos et al., 2015). In the same context blocking of dopamine D_2_ receptors attenuated morphine antinociceptive tolerance in mice (Dai et al., 2016). However, reports addressing the role of D_2_ receptors in morphine antinociception in combination therapy are scarce. One study reported that dopamine potentiated propranolol’s cutaneous analgesia (Chen et al., 2015). The observed attenuation of the antinociceptive response of propranolol-morphine combination by haloperidol in our study implicates the activation of dopamine receptors as a possible antinociceptive mechanism of the combination. There is a cooperative pathway between opioidergic and dopaminergic effects (Wood, 1983; Nestler, 1996). Furthermore blocking of the D_2_ receptors has been shown to prevent the inhibitory effect of dopamine on AC enzyme and the reduction of cAMP level (Rangel-Barajas et al., 2015) which in turn would antagonize the inhibitory effect of morphine and propranolol on reducing cAMP level and reversed their antinociceptive effect. The involvement of downstream signaling cascade could explain the potentiating effect of morphine-propranolol combination observed in the performed pharmacological studies. A proposed mechanism of the crosstalk between opioid, β-adrenergic and dopamine receptors is presented in **Figure 5**.

It is important to comment on the differential effect of haloperidol on the antinociceptive effect of combined propranolol-morphine regimen. Haloperidol antagonized the antinociceptive effect of propranolol-morphine only in the first phase of the formalin test. Similarly, sulpiride another D_2_ receptor antagonist reversed the antinociceptive effects of morphine only in the first phase of formalin-induced orofacial pain in rats (Reisi et al., 2014). The failure of haloperidol to attenuate the antinociceptive effect of propranolol-morphine in the second phase of formalin test is difficult to explain. Possible justification to this observation may be that the nociceptive component of the first phase of the formalin test reflects acute neurogenic origin that involves the Aδ fibers. However, direct stimulation of chemical nociceptors triggered the input from C fibers, which in turn provokes the second inflammatory response (Pajot et al., 2000). Therefore, it is conceivable that the differential implication of dopamine receptors in the antinociceptive response of propranolol-morphine during the two phases of formalin test may be attributed to the different nociceptive stimuli or the different origin of nerve input within the pain model itself. Similar conclusions were previously reported for morphine (Afify et al., 2013), khat extract (Afify et al., 2017), amphetamine (Clarke and Franklin, 1992), and nicotine (Damaj, 2007).

Other mechanisms, however, may contribute to the analgesic effect of the combination of propranolol with morphine in pain management. In the present study, bicuculline, a GABA_A_ receptor antagonist, blocked the antinociceptive response of propranolol-morphine therapy in the hot plate and acetic acid models of nociception. The vast distribution of GABAergic neurons throughout the peripheral and central nervous system established the role of GABA in transmission and perception of pain impulses (Carlton et al., 1999). For example, the GABA_A_ receptor agonists (Carlton et al., 1999; Motta et al., 2004), and the GABA-mimetic drugs (Carlton and Zhou, 1998) induced peripheral antinociception in the formalin test. Remarkably, β-agonists decreased GABA activity in isolated preparations through the activation of cAMP and PKA (Danielsson et al., 2016) whereas the β-adrenergic blocker, propranolol can stimulate GABA benzodiazepine receptor coupling (Benistant et al., 1988). Thus the current results indicated that propranolol by blocking the β-receptors and negatively modulates cAMP activity may increase the release of GABA and augments the antinociceptive action of morphine. This action is blocked by the GABA_A_ antagonist, bicuculline. Taken together, both gabaergic and dopaminergic receptors are involved, at least in part in the antinociceptive effect of propranolol-morphine combination.

We further investigated the effect of repeated propranolol administration on morphine antinociception. Administration of propranolol for 4 days potentiated morphine analgesia in the three nociceptive tests. In the inflammatory model, the potentiation was only noticeable in the second phase of the formalin test. It is well-established that the direct stimulation of nociceptors triggers inflammatory response in the late phase of formalin test (Pajot et al., 2000) that caused by the released cytokines and inflammatory mediators (Oprée and Kress, 2000). This in turn stimulates C-fibers (Ringkamp et al., 2011) and the nociceptive transmission in the second phase of formalin test. Propranolol and other β-blockers (Benish et al., 2008; Nguyen et al., 2008; Kato et al., 2009) abrogate the proinflammatory cytokines (Deten et al., 2003; Tang et al., 2008) and exert an anti-inflammatory response. Propranolol decreased the T helper type 1 cytokine profile in human leukemic T cells (Hajighasemi and Mirshafiey, 2016). Moreover, it inhibits the proliferative activity and the vascular endothelial growth factor production on peripheral blood mononuclear cells (Hajighasemi and Mirshafiey, 2010). Not surprisingly, chronic propranolol administration via its anti-inflammatory effect significantly potentiated morphine antinociception during the inflammatory second phase of formalin test.

## Conclusion

The current study is the first to report on the antinociceptive mechansim of morphine-propranolol treatment. The inhibition of the antinociceptive effect of morphine-propranolol therapy in the presence of the GABA_A_ blocker suggests a role for gabergic receptors in the antinociceptive effect. The inhibtion of dopamine receptor is another mechanism that might contribute to propranolol-morphine analgesic effect. Moreover, the antinociceptive effect of the combined propranolol-morphine regimen depends on the nature of the painful stimulus and the activated nerve fiber. The results suggest that propranolol may be useful as a new alternative add on therapy to morphine for controlling pain. More mechanistic studies are required, however, to elucidate the possible crosstalks between opioids, adrenergic and other receptors in modulating pain transmission. Interpretation of results of this work is expected to open new avenues for improving the efficacy of opioids in management of pain.

## Disclosure

The submitted paper was orally presented at the ESPET 56th Annual Conference and Workshop on Behavioral pharmacology held at the British University in Egypt (BUE) at El-Shorooq City on January, 28th 2017. The abstract was published in “booklet Abstracts” in the ESPET conference manual. The actual manuscript, however, has not been submitted or published in any journal.

## Ethics Statement

All applicable international, national, and/or institutional guidelines for the care and use of animals were followed.

## Author Contributions

EA raised the idea, designed the experimental protocol, data analysis and interpretation of the results, manuscript preparation and work overseeing. NA performed the experiments and collected data.

## Conflict of Interest Statement

The authors declare that the research was conducted in the absence of any commercial or financial relationships that could be construed as a potential conflict of interest.
